# An Initial Assessment of Rabbit Cornea as a Biomarker of Trace-Element Load in Commercial Animal Production

**DOI:** 10.3390/metabo16030177

**Published:** 2026-03-07

**Authors:** Nikita Filatov, Marina Kravchik, Airat Bilyalov, Ivan Novikov, Angelina Titova, Stepan Perepechenov, Olga Pak, Anastasia Novikova, Khusam Khraistin, Alexandra Karunas, Oleg Gusev

**Affiliations:** 1 Life Improvement by Future Technologies (LIFT) Center, 121205 Moscow, Russia; 2 Institute of Biochemistry and Genetics, Ufa Federal Research Center of the Russian Academy of Sciences, 450054 Ufa, Russia; 3 Krasnov Research Institute of Eye Diseases, 119021 Moscow, Russia; 4 Moscow Clinical Scientific Center Named After Loginov, 111123 Moscow, Russia; 5 Institute of Fundamental Medicine and Biology, Kazan Federal University, 420008 Kazan, Russia; 6 Intractable Disease Research Center, Graduate School of Medicine, Juntendo University, Tokyo 113-8421, Japan

**Keywords:** corneal tissue, trace-element load, copper and iron metabolism, biomarker development, atomic absorption spectroscopy

## Abstract

**Background/Objectives:** Assessing trace-element status is fundamental for maintaining health across species. However, serum primarily reflects acute physiological variability rather than chronic exposure. Thus, we investigate the cornea as a possible stable, practical alternative for assessing chronic copper and iron accumulation in rabbit’s cornea. **Methods:** A group of laboratory rabbits was housed under standardized husbandry conditions with comparable environmental and dietary backgrounds for trace-element intake. After completion of the experimental phase, corneal tissues were collected and subjected to quantitative elemental analysis using validated spectrometric procedures. In parallel, the structural integrity of the cornea was evaluated with standard histological techniques to determine whether elevated trace-element levels were associated with detectable morphological alterations. **Results:** Copper and iron concentrations showed approximately normal distributions, with mean values of 0.93 ± 0.46 μg/g and 0.78 ± 0.32 μg/g. All elemental concentrations were calculated relative to the original (native) wet tissue weight. Several samples exhibited elevated levels of both elements. Importantly, even in the samples with the highest copper and iron concentrations, no histological abnormalities were observed. Epithelial layers were intact, stromal collagen was well organized, and no inflammation or edema was observed. **Conclusions:** Overall, the cornea contained measurable copper and iron levels, and higher concentrations were not associated with morphological disruption under the trace-element conditions studied. Because ocular tissues are not used in food processing and can be collected in a standardized way during slaughter, the cornea offers a practical matrix for post-mortem monitoring of trace-element load in commercial animal production.

## 1. Introduction

Modern industrial livestock production requires precise tools for assessing the metabolic state of animals. Monitoring trace-element status has become an important component in preventing deficiencies and toxic effects that can impair growth, reproductive performance, immune function, and the overall biological potential of the herd [1,2]. The practical value of such tools lies in the early detection of deviations and in the ability to adjust feeding rations before clinically significant disorders develop.

Trace elements such as iron, copper, zinc, and selenium are essential for ocular metabolism and antioxidant defense, and disturbances in their homeostasis contribute to the pathogenesis of several degenerative eye diseases [3,4]. Recent metallomic studies indicate that the cornea, aqueous humor, lens, and retina function as an integrated system in which shifts in trace-element balance manifest as tissue-specific patterns of distribution and accumulation [5,6]. Quantitative elemental analyses in humans and experimental animals further show that calcium, copper, iron, and zinc occur in ocular tissues in a nonuniform and species-specific manner, with distinct distributions across the cornea, lens, iris, and retina; notably, paired eyes from the same individual often exhibit closely matching elemental profiles [3,6]. Together, these findings support the view that ocular tissues can contain measurable trace-element pools and may provide information complementary to serum markers, which can be influenced by short-term physiological variability.

Among ocular structures, the cornea appears particularly promising for assessment of trace-element status. The cornea is a transparent, multilayered tissue composed of an outer epithelium, Bowman’s layer, a thick collagen-rich stroma, Descemet’s membrane, and an inner endothelium. The stroma accounts for most of the corneal thickness and is formed by regularly arranged collagen lamellae with keratocytes embedded between them [7]. Being avascular, the cornea receives most solutes by diffusion rather than direct perfusion. Key sources include the aqueous humor, the tear film, and the pericorneal (limbal) circulation, while metabolic end products are removed via the aqueous humor, tears, and limbal vasculature [8]. This physiology makes it plausible that trace-metals present in ocular fluids can enter corneal tissues and form measurable tissue pools. Clinical entities such as Kayser–Fleischer rings in Wilson disease and Fleischer rings in keratoconus illustrate the capacity of corneal tissues to accumulate copper and iron to levels detectable by routine ophthalmological examination [9,10]. In Wilson disease, excess copper can deposit in the peripheral cornea within the Descemet membrane, and proposed mechanisms include entry from the aqueous humor across the endothelium followed by binding within basement membrane-associated structures [9]. Iron can also accumulate in the corneal epithelium as clinically visible iron lines, and histopathology has identified ferritin particle–associated iron within basal epithelial cells, with formation linked to tear film dynamics and local epithelial turnover rather than systemic perfusion [11]. Although these observations do not define the kinetics of accumulation, they substantiate the biological feasibility that corneal compartments can receive and retain metal species in ways that may not be fully detected by instantaneous systemic measurements. Consistent with this, ocular fluids contain measurable trace metal concentrations, including zinc, iron, and copper in the aqueous humor, which provides a physiologically relevant reservoir for diffusion into the avascular cornea [12]. In this study, an alternative approach to assessing the trace-element status of animals is proposed by determining copper and iron levels in the cornea using atomic absorption spectroscopy [13,14]. This approach makes it possible to identify patterns of long-term accumulation of elements in tissues that are not subject to rapid metabolic fluctuations [15]. Copper and iron were selected as the test elements. Both metals exhibit variable valence states and are transported primarily as components of metalloprotein complexes, from which they are subsequently deposited in tissues [16]. In this study, the cornea is examined as a promising tissue for evaluating chronic trace-element accumulation. Because it is an avascular structure that does not respond to fluctuations associated with transient trace-element influx through the bloodstream, it provides a smoothed accumulation profile that reflects prolonged exposure [15]. An additional advantage is that eyes are not used in food processing and can be collected in a standardized manner after slaughter, which improves reproducibility and simplifies the practical application of the method. Given the similarity of housing and feeding conditions in rabbit and poultry production, rabbits can be considered a partially comparable model for extrapolating the results to agricultural poultry. The aim of this study is to evaluate the informational value of the cornea as a biological substrate for monitoring copper and iron accumulation in rabbits raised under conditions comparable to industrial poultry production.

## 2. Materials and Methods

### 2.1. Design and Conditions of the Experimental Study

The study included Soviet Chinchilla laboratory rabbits (n = 22), clinically healthy and aged 4–6 months, which corresponds to the physiologically mature stage for this breed and ensures the representativeness of the experimental model. Animal sex was not considered a variable, as the experimental protocol and the presumed mechanism of passive diffusion of trace elements into the corneal stroma do not suggest any significant influence of sex differences on pharmacokinetic parameters [16,17].

Animals were housed in a certified vivarium in accordance with current veterinary and sanitary regulations. All animals were fed a commercial pelleted complete diet for rabbits that complies with the Russian national standard GOST 32897 [18]. This standard regulates permissible concentrations of several trace elements, including copper, which must not exceed 30–80 mg per kg of feed. Although our study focuses on copper and iron, only copper is specified in the standard, while iron levels are not regulated.

Prior to inclusion in the study, all individuals underwent a clinical examination by a veterinary specialist to rule out ocular pathology and systemic diseases. Based on the examination results, all animals were deemed eligible for participation. A 14-day quarantine period was observed in accordance with standard operating procedures. Only rabbits with no abnormalities in daily health status or digestive function were enrolled in the experimental phase. Animal husbandry conditions were fully standardized, and rabbits were housed individually in cages with bedding, had ad libitum access to drinking water, and were provided with standard pelleted laboratory feed. Environmental parameters in the vivarium were maintained within regulated limits, including an air temperature of 18–22 °C, relative humidity of 40–60%, and a 12 h light/dark cycle. Animals were monitored daily by veterinary staff.

For biological sample collection at the predefined experimental endpoint, humane euthanasia was performed using carbon dioxide (CO_2_) in a dedicated chamber, in accordance with the approved protocol [19]. In the context of this study, corneal sampling was carried out exclusively at this endpoint, and no animals were euthanized specifically for the purposes of corneal analysis. Following euthanasia, enucleation and dissection of the eyeball were performed. The cornea was excised as an intact full-thickness flap by circumferential dissection along the limbus, with a safety margin, avoiding direct contact between metallic instruments and the corneal tissue. The isolated cornea was then sectioned and processed for morphological and chemical analyses. In practical applications, the proposed approach is intended for post-mortem sampling during routine slaughter or planned culling in commercial production systems. The experimental unit was the animal, and only one eye per rabbit was collected and processed for all analyses.

All animal experiments were approved by the bioethics committee of the national medical research center of cardiology named after academician E. I. Chazov (ethical approval certificate No. EBK/26.09.25 dated 30 September 2025).

### 2.2. Analysis of the Elemental Composition of the Cornea

#### 2.2.1. Preparation of Corneal Samples for Chemical Analysis

The elemental composition of the cornea was analyzed to quantitatively determine copper and iron levels accumulated. For trace-element quantification, the entire excised cornea was processed. Stromal material was obtained from the full corneal tissue rather than from a thickness-standardized lamellar dissection, and the same full-corneal workflow was applied to all samples.

Isolated corneas were dissected to isolate the stromal component. The stromal layer was selected for analysis because it represents an avascular and structurally stable matrix for trace-element quantification, thereby minimizing variability associated with vascularized tissues [20]. To minimize the risk of metallic contamination, a sapphire blade was used. Each sample was weighed in its natural hydration state for subsequent concentration calculations. The tissue was transported to the analytical laboratory in individual airtight containers at −5 °C. Upon arrival, samples were frozen at −60 °C and lyophilized under 10 Pa pressure for 1.5 h. After drying, samples were reweighed and subjected to chemical digestion using a nitric acid protocol combined with ashing in hydrogen peroxide, performed in two stages over a 24 h period.

Nine parts by weight of 70% nitric acid (analytical grade, Komponent-Reaktiv, Moscow, Russia) were added to the dry mass of each sample. The decomposition was carried out at room temperature in open quartz dishes under a fume hood for 24 h until complete digestion of the organic phase. An additional ten parts by weight of the same acid (relative to the initial dry sample mass) were then added.

The resulting solution was evaporated on a hot plate at 200–240 °C for 10–20 min until complete removal of the liquid. The presence of residual acid and organic impurities was monitored by adding a 10 μL drop of 37% hydrogen peroxide (Khim-Baza, Moscow, Russia). After cooling, the solution was brought to a final volume of 1 mL using HPLC-grade water (Komponent-Reaktiv) [21,22]. The prepared solutions were transferred to sample tubes and placed in the autosampler of the atomic absorption spectrometer.

#### 2.2.2. Determination of Copper and Iron Content by Atomic Absorption Spectroscopy

Quantitative determination of copper and iron content in the corneal stroma was carried out using atomic absorption spectroscopy with preliminary calibration using standard solutions. Certified reference materials were used as standards: copper—GSO 7836–2000 (mass concentration 1.00 g/dm^3^, ECROCHIM, Moscow, Russia) and iron (III)—GSO 7835–2000 (1.00 g/dm^3^, ECROCHIM). Calibration series were prepared by stepwise dilution of the standard solutions with HPLC-grade water (Komponent-Reaktiv).

Measurements were performed on atomic absorption spectrometer (KVANT.Z, KORTEK LLC, Moscow, Russia) using an automatic sample feeder. The Zeeman-corrected analytical absorbance signal A was recorded for each calibration solution and used to construct calibration curves, using the peak amplitude of the absorbance pulse as the primary analytical parameter. The signal level for each calibration solution is presented in Table 1. Instrument control and data acquisition were carried out using the proprietary KVANT.Z software (version 3.0.0, build 3.5.8).

The concentrations of copper and iron for each specimen, copper and iron concentrations were determined from five replicate measurements, followed by calculation of the mean value and standard deviation. These repeated measurements represented technical replicates used solely for analytical quality control and were not treated as independent observations in statistical analyses, which were conducted at the level of biological replicates. The instrumental results (C, µg/dm^3^) were adjusted according to the mass of the original (wet) sample and all changes occurring during sample preparation, including reagent addition and potential losses, ensuring the accuracy of quantitative assessment of the corneal tissue’s trace-element composition. This allowed calculation of native corneal tissue concentrations expressed on a wet-weight basis (µg/g).

### 2.3. Quality Assurance and Quality Control (QA/QC)

Full-procedure blanks were prepared and analyzed using the complete digestion protocol (HPLC-grade water, nitric acid, hydrogen peroxide; open-vessel digestion and evaporation) without tissue (n = 5). Mean instrumental full-procedure blank concentrations (±SD) were 0.86 ± 0.28 mg/L for copper and 3.27 ± 0.83 mg/L for iron.

In addition, blank water measurements (HPLC-grade water) (n = 5) were per-formed. Mean instrumental HPLC-grade water blank concentrations (±SD) were 0.94 ± 0.36 mg/L for copper and 1.24 ± 0.76 mg/L for iron.

Calibration was performed using certified reference solutions (GSO 7836–2000 for copper and GSO 7835–2000 for iron (III)). Calibration curve parameters (slope, intercept, and coefficient of determination, R^2^) were as follows: for copper (0–40 µg/L), slope = 0.0087, intercept = 0.0097, R^2^ = 0.995; and for iron (0–100 µg/L), slope = 0.0057, intercept = 0.168, R^2^ = 0.957

Limits of detection (LOD) and quantification (LOQ) were estimated from full-procedure blanks using meanf-p_blank + 3σ and meanf-p_blank + 10σ criteria, respectively, yielding LOD/LOQ values of 1.71 mg/L and 3.7 mg/L for copper, and 6.07 mg/L and 12.61 mg/L for iron.

### 2.4. Morphological Analysis

Morphological examination of the cornea was performed to assess whether variation in corneal copper and iron levels was associated with detectable tissue alterations. The obtained samples were fixed in 10% neutral formalin prepared on 0.2 M phosphate buffer (pH 7.4) for 24 h. Following fixation, the tissues were embedded in paraffin using the HP300 automated tissue processor (Dakewe, Shenzhen City, China). Paraffin sections 4 μm thick were prepared using a Microm HM 366S rotary microtome (Thermo Scientific, Waltham, MA, USA). The sections were then incubated in a thermostat at 37 °C for 24 h. Histological staining was performed using general-purpose dyes: hematoxylin and eosin, Mallory’s trichrome, and the periodic acid–Schiff (PAS) reaction set for glycogen detection using standardized commercial kits (ErgoProduction, Biovitrum, Moscow, Russia) according to the manufacturers’ manual. Digital histotopograms were obtained using a NanoZoomer S60 Digital Slide Scanner (Hamamatsu, Shizuoka, Japan) and analyzed using NDP.view2 software, version 2.9.29 (Hamamatsu, Shizuoka, Japan).

#### 2.4.1. Hematoxylin and Eosin Staining

Mayer’s hematoxylin and a 1% aqueous solution of eosin were used for staining. Following deparaffinization and rehydration, the sections were incubated in hematoxylin for 60 s and then differentiated in running tap water for 5 min. Eosin solution was then applied for 30–60 s, followed by rinsing in distilled water and mounting in glycerol-gelatin. In hematoxylin- and eosin-stained sections nuclei appear dark blue to violet and the cytoplasm and stromal collagen show different shades of pink. This contrast allows reliable assessment of the general corneal architecture, cellularity, edema and inflammatory cell infiltration.

#### 2.4.2. Mallory’s Trichrome Staining

Staining was performed according to the manufacturer’s instructions for the Mallory staining kit (BioVitrum, Moscow, Russia). After deparaffinization, the sections were incubated in carbol fuchsin for 10 min and rinsed in distilled water. A phosphomolybdic acid solution was applied for 5 min following a 2 min treatment with an acid buffer and brief rinsing in running water. Mallory’s polychrome solution was applied for 1 min, followed by a rinse in distilled water. The sections were then dehydrated through graded alcohols, cleared in xylene, and mounted in polystyrene (BlikMedicalProduction, Taganrog, Russia). In Mallory trichrome stained sections stromal collagen fibers are visualized in bright blue tones, whereas the cytoplasm of stromal and endothelial cells appears in red hues and nuclei are dark blue. This pattern highlights the organization and density of the collagen matrix and facilitates the detection of fibrotic or disorganized stromal areas.

#### 2.4.3. Periodic Acid–Schiff (PAS) Reaction

Staining was carried out following the instructions for the PAS staining kit (BioVitrum, Moscow, Russia). After deparaffinization, the sections were incubated in periodic acid solution for 5 min, rinsed in distilled water, and treated with Schiff’s reagent for 20 min. A subsequent rinse in distilled water was performed. Counterstaining was done with Mayer’s hematoxylin, followed by rinsing in tap water and mounting in glycerol-gelatin. In sections processed with the periodic acid–Schiff reaction PAS positive structures such as epithelial basement membrane and glycoprotein rich layers are stained in magenta while nuclei are counterstained in blue. This combination provides clear visualization of the basement membrane zone and other carbohydrate rich components that are relevant for the assessment of corneal integrity.

## 3. Results

### 3.1. Chemical Analysis

#### 3.1.1. Detection of Abnormal Iron and Copper Concentrations in the Samples

To initially identify individual markedly abnormal observations in the sample, z-score analysis was applied, representing the standardized deviation of a value from the sample mean. An empirical threshold of |z| > 3 was used. Based on this criterion, one clearly extreme iron concentration value was identified: sample No. 14, iron = 6.64 µg/g, corresponding to a z-score of 4.35. No copper values exceeded |z| > 3. In subsequent analysis, descriptive statistics were calculated after excluding the identified iron outlier. Thus, copper analysis was performed on 22 observations, and iron on 21. Descriptive statistical analysis and graphical visualization were performed using R with the ggplot2 package.

#### 3.1.2. Distribution of Copper Concentrations in Rabbit Corneas

**Figure 1:**
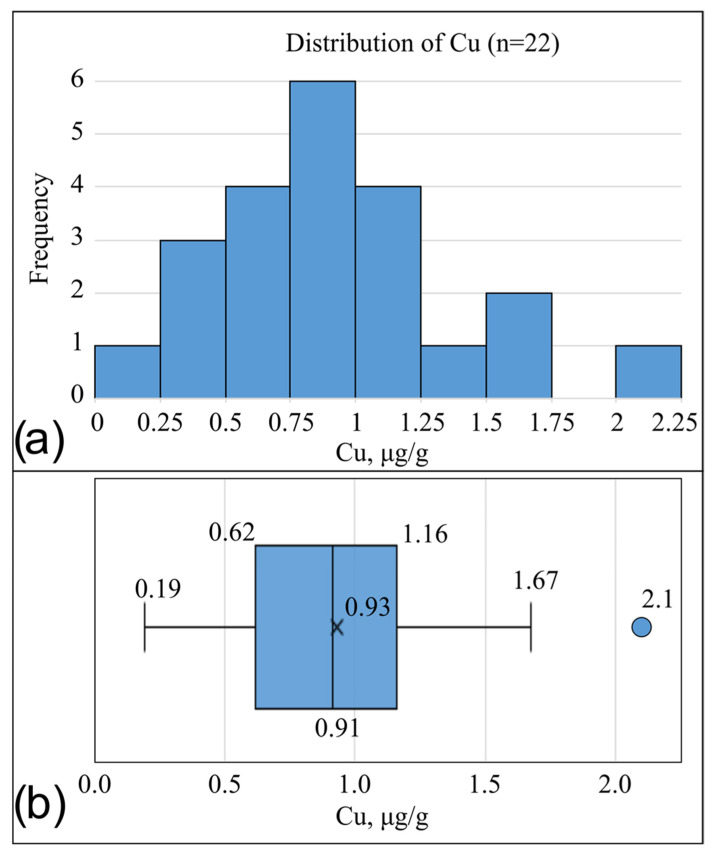
Distribution plots of copper (copper) concentrations in the cornea of laboratory rabbits. Values are expressed in µg/g (native wet weight). (**a**)—histogram of distribution; (**b**)—boxplot showing the interquartile range and outliers. A near-normal distribution and a single outlier are observed.

In the analyzed sample (n = 22), the distribution of copper concentrations (copper, µg/g) approximated a normal distribution, allowing statistical processing using parametric methods (Figure 1a). The skewness coefficient was 0.612, indicating moderate and acceptable right-sided asymmetry. The kurtosis value of 0.27 indicates no significant deviations from normality.

On the boxplot diagram (Figure 1b), the median was symmetrically located within the interquartile range, and one moderate outlier was recorded—sample No. 4, with a copper concentration of 2.10 µg/g. This result qualified as an outlier under the interquartile range (IQR) rule, exceeding Q3 + 1.5 × IQR.

Collectively, the parameters listed above allowed the distribution of copper concentrations to be considered approximately normal. Therefore, the mean and standard deviation were used to describe the central tendency (Table 2).

#### 3.1.3. Distribution Characteristics of Iron Levels in Rabbit Corneas

**Figure 2:**
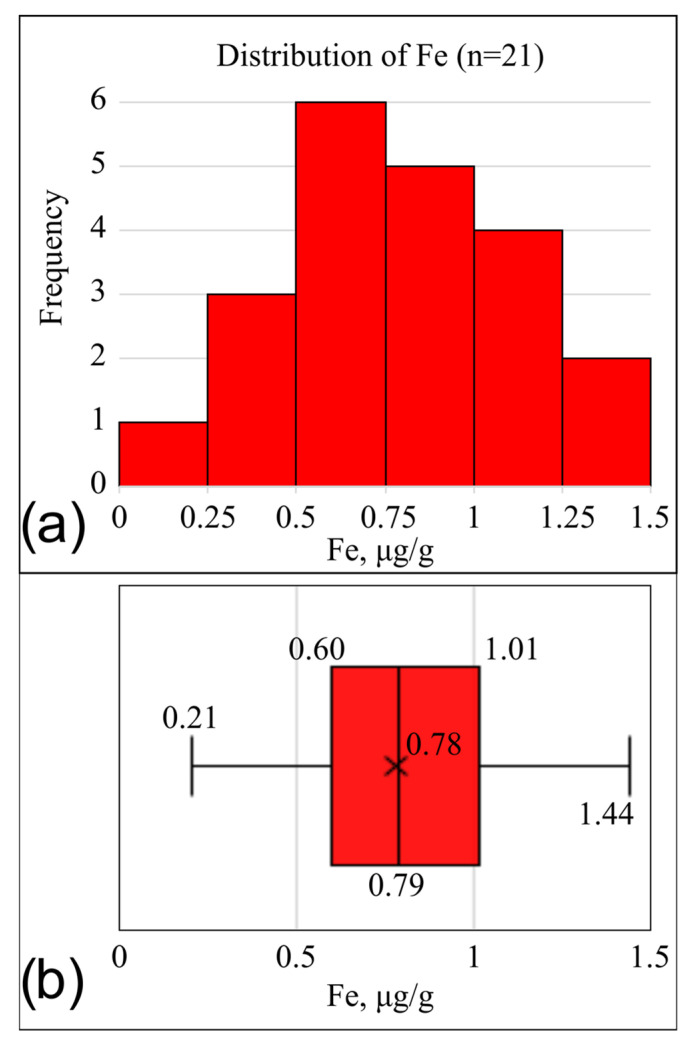
Distribution plots of iron (iron) concentrations in the cornea of laboratory rabbits after exclusion of the extreme outlier. Values are expressed in µg/g (native wet weight). (**a**)—histogram of distribution excluding the extreme value; (**b**)—boxplot excluding the extreme value. The distribution shows slight asymmetry and approximates normality. The full dataset, including the outlier, is presented in Supplementary Figure S1.

The analysis of iron (iron, µg/g) concentrations was performed on a subsample excluding the previously identified extreme value (n = 21). In this group, the distribution was also close to normal (Figure 2). The skewness coefficient was 0.27, indicating mild right-sided asymmetry. The kurtosis coefficient was −0.441, suggesting a slight flattening of the distribution compared to the normal curve.

The combination of these parameters allowed the distribution of iron concentrations to be considered symmetric. Therefore, the mean and standard deviation were used to describe central tendency and dispersion (Table 2).

### 3.2. Histological Characteristics of the Cornea

#### 3.2.1. Corneal Structure in Animals Without Signs of Trace-Element Accumulation

**Figure 3:**
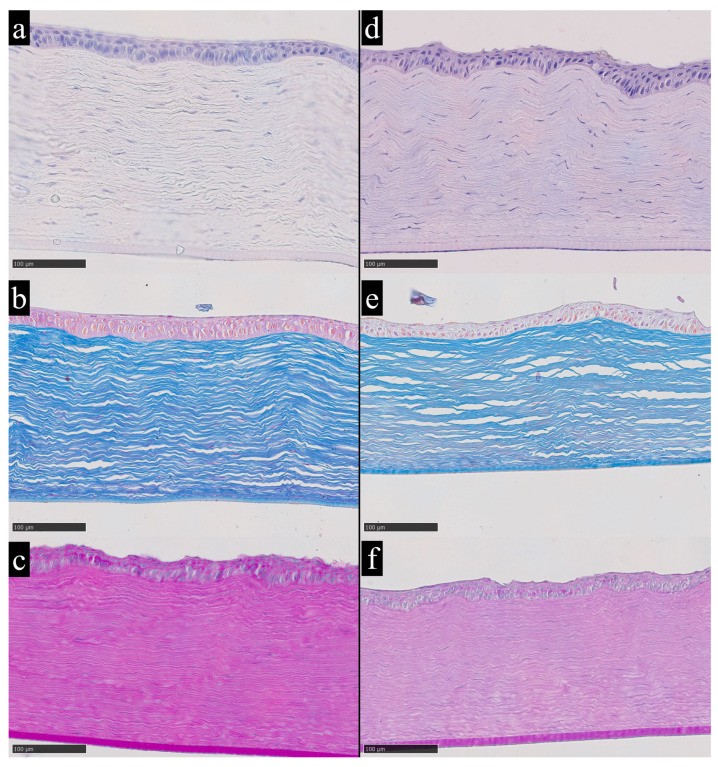
Cornea of a rabbit without morphological alterations and without elevated trace-element concentrations (**a**–**c**) and cornea of rabbit No. 14 with elevated iron content according to chemical analysis and without pronounced morphological changes (**d**–**f**); (**a**,**d**)—hematoxylin and eosin staining, (**b**,**e**)—Mallory’s staining, (**c**,**f**)—PAS staining. Hematoxylin and eosin provide an overview of corneal architecture and cellularity, with nuclei staining blue to violet and cytoplasm and stroma staining pink. Mallory’s trichrome highlights stromal collagen distribution and organization, with collagen fibers staining blue and cytoplasm staining red. Periodic acid–Schiff staining highlights carbohydrate rich structures, including basement membrane associated glycoconjugates, with PAS positive material staining magenta. Magnification ×200.

In the experimental animals whose corneas showed no evidence of copper or iron accumulation based on elemental analysis, the histological structure corresponded to normal morphological criteria (Figure 3a–c). Stratified non-keratinized squamous epithelium retains its typical architecture. The corneal stroma shows parallel alignment of collagen lamellae without signs of disorganization. Keratocytes are present in a normal quantity, with typical morphology and uniform distribution between collagen layers. No inflammatory infiltration or vascular structures are observed. Overall tissue transparency is within normal limits, as evidenced by uniform staining and the absence of edema.

#### 3.2.2. Morphological Features of the Cornea in Cases of Copper and Iron Accumulation

As previously noted, the analysis of trace-element concentrations in the corneal stroma revealed isolated instances of significant deviations beyond background variability. The most pronounced deviation in iron content was observed in the corneal sample from rabbit No. 14, which, according to the z-score criterion, qualified as a statistically significant outlier relative to the entire dataset (Figure 3d–f).

**Figure 4:**
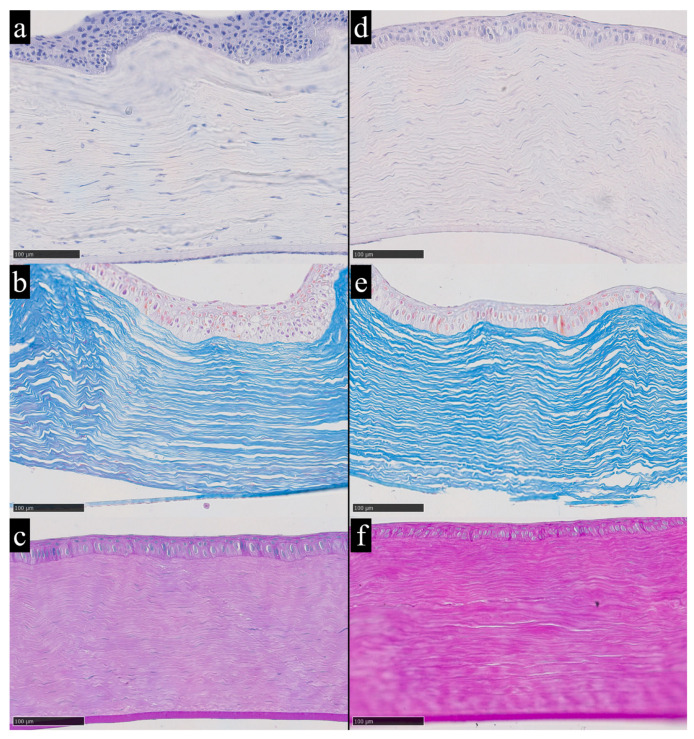
Corneas of rabbits No. 1 (**a**–**c**) and No. 18 (**d**–**f**) with elevated iron content according to chemical analysis and without significant morphological changes; (**a**,**d**)—hematoxylin and eosin staining; (**b**,**e**)—Mallory’s method; (**c**,**f**)—periodic acid–Schiff (PAS) staining. Hematoxylin and eosin provide an overview of corneal architecture and cellularity, with nuclei staining blue to violet and cytoplasm and stroma staining pink. Mallory’s trichrome highlights stromal collagen distribution and organization, with collagen fibers staining blue and cytoplasm staining red. Periodic acid–Schiff staining highlights carbohydrate rich structures, including basement membrane-associated glycoconjugates, with PAS positive material staining magenta. Magnification ×200.

In addition to the clearly extreme case, observations on the morphological features of the cornea under conditions of elevated iron content were based on samples with the highest recorded iron concentrations among the remaining dataset. In particular, rabbits No. 1 and No. 18 showed stromal iron levels of 1.27 µg/g and 1.44 µg/g, respectively. These samples exhibited conditionally high levels of trace-element load, placing them in the upper part of the distribution (Figure 4).

**Figure 5:**
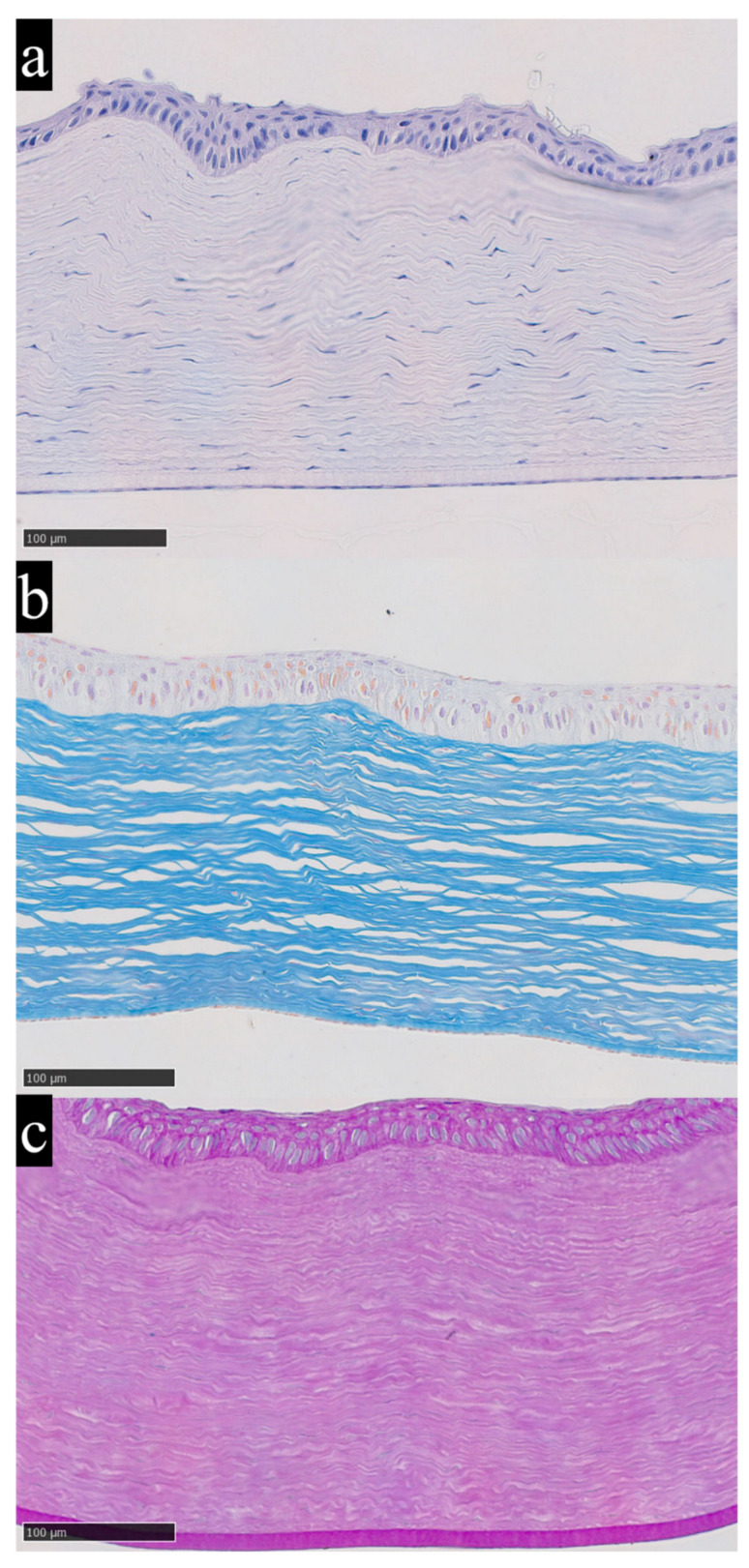
Cornea of rabbit No. 4 with a moderate increase in copper content based on chemical analysis, with no significant morphological alterations observed. (**a**)—hematoxylin and eosin staining; (**b**)—Mallory staining; (**c**)—PAS staining. Hematoxylin and eosin provide an overview of corneal architecture and cellularity, with nuclei staining blue to violet and cytoplasm and stroma staining pink. Mallory’s trichrome highlights stromal collagen distribution and organization, with collagen fibers staining blue and cytoplasm staining red. Periodic acid–Schiff staining highlights carbohydrate rich structures, including basement membrane associated glycoconjugates, with PAS positive material staining magenta. Magnification ×200.

The distribution of copper concentrations showed a different pattern compared to iron. When the interquartile range method (1.5 × IQR) was applied, a moderate outlier was detected. It corresponded to the corneal sample from animal No. 4, which had a copper concentration of 2.10 μg/g (Figure 5).

**Figure 6:**
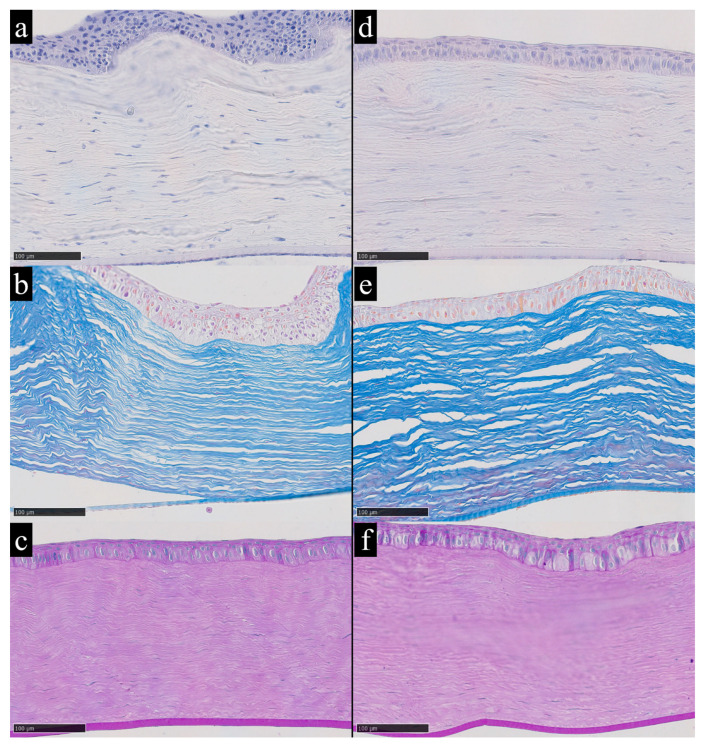
Corneas of rabbits No. 1 (**a**–**c**) and No. 2 (**d**–**f**) with elevated copper content according to chemical analysis and without pronounced morphological changes; (**a**,**d**)—hematoxylin and eosin staining; (**b**,**e**)—Mallory staining; (**c**,**f**)—PAS staining. Hematoxylin and eosin provide an overview of corneal architecture and cellularity, with nuclei staining blue to violet and cytoplasm and stroma staining pink. Mallory’s trichrome highlights stromal collagen distribution and organization, with collagen fibers staining blue and cytoplasm staining red. Periodic acid–Schiff staining highlights carbohydrate rich structures, including basement membrane associated glycoconjugates, with PAS positive material staining magenta. Magnification ×200.

Histological evaluation of the corneal stroma under elevated copper levels was based on samples with the highest copper concentrations among the remaining observations. In rabbit No. 1, the copper concentration reached 1.67 μg/g, and this same sample also showed increased iron content, as described earlier. In rabbit No. 2, the copper level was 1.51 μg/g. These values fell within the upper range of the sample distribution and were considered conditionally elevated (Figure 6).

In all samples with elevated copper and iron levels identified by chemical analysis, the corneal morphology remained within normal limits. The epithelium was preserved and clearly stratified, with no evidence of stromal edema or inflammatory infiltration. Collagen lamellae displayed uniform staining, interlamellar spaces were not widened, and both Descemet’s membrane and the endothelium appeared unremarkable.

## 4. Discussion

Several lines of evidence support the biological plausibility of using the cornea as a trace-element matrix. The concept of this study is based on the fundamental characteristics of the cornea as an avascular structure, which minimizes its susceptibility to short-term fluctuations in systemic trace-element levels and may contribute to the formation of a stable pattern of accumulation [8,11,23]. This property makes the cornea a promising analytical substrate in industrial animal production, where a reliable tool for assessing trace-element load is required. Quantitative elemental mapping in the mammalian cornea shows that endogenous metals are distributed non-homogeneously and that iron and copper can be detected with preferential localization, particularly within epithelial layers, with iron also identified in stromal keratocytes [24]. Copper is measurable in corneal tissue across multiple vertebrate species, including rabbits, using atomic absorption-based assays, indicating that corneal copper levels fall within a quantifiable range suitable for analytical workflows [25]. Clinical and histopathological observations further indicate that iron can accumulate in the corneal epithelium, as exemplified by Fleischer ring-like pigmentation attributed to hemosiderin deposition [20]. From a tissue-architecture standpoint, the corneal stroma is a collagen-rich extracellular matrix with highly ordered lamellae, providing a stable structural scaffold that can, in principle, retain trace-element signatures within the stromal microenvironment [26]. Corneal collagen turnover is widely considered to be relatively slow, which is consistent with the feasibility of using stromal matrices in approaches aimed at capturing integrated biological signals [27].

In commercial livestock production, early detection of non-physiological trace-element levels is essential for preventing declines in productivity and reproductive performance. Traditional assessment of trace-element status relies mainly on serum, whole blood, and internal organs, as summarized in reviews on trace-mineral evaluation and livestock nutrition [28,29]. Although informative, serum-based indicators are strongly influenced by acute-phase responses and systemic inflammation, leading to transient shifts in circulating iron indices and acute-phase proteins that complicate the interpretation of single blood measurements with respect to long-term exposure [30,31,32]. Mechanistically, circulating copper is largely associated with ceruloplasmin, and ceruloplasmin behaves as a mild-to-moderate acute-phase protein in cattle. Therefore, inflammatory states can increase ceruloplasmin activity and elevate serum copper independently of dietary copper exposure, which reduces the specificity of single time-point serum copper measurements for retrospective assessment [33,34]. Likewise, inflammation induces hepcidin, which suppresses iron export to plasma by downregulating ferroportin and produces rapid hypoferremia, making serum iron highly time-dependent [35]. In cattle, serum iron has been evaluated explicitly as an indicator of acute inflammation, underscoring the extent to which inflammatory status can dominate short-term circulating iron measurements [36]. This physiological instability reduces the usefulness of serum as a stand-alone retrospective marker of cumulative trace-element exposure. In contrast, the cornea, owing to its avascularity and general physiological characteristics, exhibits a relatively low level of metabolic activity [37], and the turnover of its stromal matrix is slower than the rapid fluctuations observed in serum so trace-elements incorporated into corneal tissue are expected to integrate exposure over longer intervals rather than individual feeding events. This view is consistent with elemental-mapping and histochemical data showing characteristic and relatively stable distribution patterns in ocular tissues [24,38]. These properties substantiate the rationale for considering the cornea as a potentially stable matrix for monitoring cumulative trace-element exposure under intensive production conditions, although direct kinetic data on corneal trace-element turnover remains limited and standardized reference ranges for corneal copper and iron in laboratory animals are unavailable. Accordingly, we interpret the present results primarily in a comparative framework within a tightly controlled cohort, and we use representative values in the literature to contextualize the order of magnitude rather than to define diagnostic thresholds. For copper, adult rabbit corneas measured by electrothermal atomic absorption spectroscopy have been reported at 0.673 ± 0.064 µg per g wet weight [25]. For iron, rabbit corneal tissue analyzed by graphite furnace atomic absorption spectrometry on freeze dried samples has been reported at 10.1 and 14.3 µg per g tissue on a dry weight basis in operated and unoperated corneas, underscoring that published values are method-dependent and influenced by normalization [39]. Under commercial husbandry conditions, rabbit and poultry production share similar parameters related to feeding regimens and housing environments. This creates a basis for limited extrapolation of experimental findings obtained in rabbit and poultry models. In particular, technological and regulatory approaches to formulating diets for rabbits and farmed poultry are largely comparable. According to GOST 18221-2018 [40] (for poultry) and GOST 32897 [18](for fur-bearing animals, rabbits, and nutria), both sectors employ standardized complete feed formulations manufactured under controlled industrial conditions, with regulated energy content, crude protein levels, moisture limits, particle structure, contaminant thresholds, and safety requirements. The main differences concern the acceptable proportion of crude fiber and the amino acid profile of the diet, which reflect species-specific digestive physiology [41,42].

In the present work, the concentrations of copper and iron measured in rabbit corneal tissue were low, varied within a relatively narrow range, and were not accompanied by microscopic evidence of stromal edema, inflammatory cell infiltration, or disorganization of the corneal stroma. These observations are consistent with published metallomic and elemental studies indicating that ocular tissues can contain measurable amounts of transition metals while maintaining normal histological architecture [3,5,6]. Clinical and experimental reports describe pronounced local enrichment of iron or copper in the cornea mainly in advanced disease, such as Wilson disease or keratoconus, when pigment deposition and marked stromal remodeling are already evident [4,8,9]. Against this background, our findings support the interpretation that the values obtained in healthy rabbits correspond to a physiological trace-element burden under intensive housing rather than to toxic accumulation, and that interindividual variation in corneal copper and iron content can occur without detectable structural damage.

Interpretation of corneal trace-element levels also requires consideration of the feeding regime used in this study. All animals received a single type of complete pelleted feed that complied with national regulations on compound feed formulation for rabbits, including established upper limits for copper content. Nutritional studies and reviews in humans and domestic animals indicate that, under such standardized conditions, tissue and whole-body trace-element status primarily reflects the habitual diet and sustained mineral supply rather than individual meals. These works consistently emphasize the importance of long-term mineral balance in preventing both deficiency and chronic oversupply [19,26]. Studies in sheep and other livestock species further show that, when energy intake and growth rate are controlled, sex and physiological category exert only limited influence on net requirements for copper, iron, and related elements [43,44]. Even under standardized feed formulations, trace-metal status is determined not only by dietary concentration but also by actual intake and bioavailability, both of which are modulated by the animal’s homeostatic adaptation to trace-metal supply [45]. The chemical form of trace minerals and their relative bioavailability differ substantially between inorganic and organic sources used in production feeds and supplements, which can alter the efficiency of copper and iron absorption and their subsequent distribution among body compartments under otherwise similar rations [46]. For copper in particular, livestock studies emphasize that supplementation strategies must balance adequacy against the risk of excessive hepatic loading, demonstrating that tissue deposition can increase under sustained oversupply even in the absence of overt clinical toxicity [47]. In rabbits, dietary copper supplementation has been shown to markedly increase hepatic copper concentrations, indicating that sustained differences in net copper intake can translate into measurable tissue-level changes [48]. Similarly, controlled feeding studies in Rex rabbits report that graded dietary iron supplementation increases body iron stores and alters circulating iron-related indices, supporting the expectation that prolonged iron supply influences systemic status and tissue distribution [49]. Therefore, to improve the interpretability of corneal measurements as indicators of nutritional trace-element exposure, future validation studies should quantify copper and iron concentrations in specific feed batches and water sources, document the form and dose of mineral supplements, and, where feasible, record individual- or pen-level feed intake. This would allow corneal values to be linked to net mineral intake rather than to feed formulation alone [45].

Cross-species surveys of vertebrate eyes demonstrate that calcium, copper, iron, and zinc concentrations, as well as their distribution across ocular tissues, differ between mammals and birds, supporting the view that baseline ocular mineral profiles are species dependent [50]. Nutrient requirement frameworks for poultry report copper and iron needs in mg/kg of diet and emphasize variation across production classes, reflecting species-specific physiology and growth intensity [51]. In broilers, published recommendations and industry guidance for supplemental iron span a wide range, indicating that baseline dietary exposure can vary substantially between production systems and premix strategies even within a single species [6]. In rabbits, practical recommendations for dietary copper inclusion likewise vary with physiological state and management context, suggesting that background intake ranges are not directly comparable to poultry diets [52]. Accordingly, corneal concentration ranges and decision thresholds should be interpreted relative to species-specific dietary reference values and supplementation practices, and calibration studies should explicitly stratify by species and production system [52,53]. According to the requirements of GOST 31657–2012 [53]“Edible Poultry By-Products. Technical Specifications,” the eyeballs of chickens, including broilers, are not classified as by-products. A similar regulation is contained in GOST 27747–2016 [54], which defines the technical specifications for rabbit meat: ocular tissues are not considered suitable for food use and are removed during slaughter. In current industrial practice, these anatomical structures, despite their potential analytical value, are simply discarded and excluded from further processing. Within the framework of the approach proposed in this study, however, the cornea can be regarded as an accessible and standardizable material for post-mortem assessment of trace-element accumulation. A major advantage of this method lies in the presence of an established regulatory basis. The Order of the Ministry of Agriculture of the Russian Federation dated 11 November 2024 No. 677 governs the handling of tissues not included in food products, thereby providing legal grounds for implementing procedures for the collection and analytical use of ocular tissues for veterinary control and research purposes. Sampling of eyes after slaughter does not involve removal of edible tissues and can be standardized within routine processing workflows at the plant. Importantly, in the approach we propose, ocular tissues are collected only as slaughter by-products and the method does not require targeted euthanasia of animals for analytical purposes because it relies on materials that are not used in food production. The preservation of normal corneal architecture across the full observed range of copper and iron concentrations in this study suggests that the cornea can retain an integrated record of exposure over time without immediate structural changing. Accordingly, corneal analysis may complement conventional matrices in surveillance programs aimed at detecting trace-element load in industrial animal husbandry.

Importantly, corneal analysis is not proposed as a universal replacement for in vivo sampling, but as a complementary post-mortem matrix that can be collected at scale without additional invasive procedures. Blood-based indicators remain appropriate for live-animal assessment. However, single time-point serum copper measurements may show limited diagnostic specificity in certain contexts and do not reliably reflect longer-term copper loading unless sampling coincides with acute events [47]. In parallel, several minimally invasive matrices have been explored for retrospective trace-element biomonitoring in production animals, including hair in cattle as an exposure biomarker [55]. In avian systems, feathers are widely used for trace-element monitoring and have been shown to accumulate multiple elements, supporting their application as a non-destructive matrix under appropriate standardization [56]. Within this framework, corneal sampling can be regarded as a practical post-mortem option that complements blood and other matrices by utilizing tissues routinely discarded during slaughter or planned culling workflows. In addition to these considerations, corneal sampling represents a specialized ophthalmic procedure that requires expert handling and is therefore most appropriate for controlled experimental studies or post-mortem surveillance settings. In its current form, the analytical workflow is resource-intensive due to the requirements for stringent contamination control, lyophilization, and laboratory-based instrumentation for trace-element quantification. These factors may limit routine implementation at the farm level and favor centralized laboratory support. Future methodological refinement and validation are expected to enable simplification of sample preparation and analytical procedures, thereby improving the feasibility and scalability of this approach.

The results obtained in the present experiment confirm that the proposed concept is functional. In several samples classified as conditionally “high-element,” chemical analysis revealed elevated levels of copper and iron, while the corneal histological structure remained within normal morphological limits. The absence of inflammatory or destructive changes despite the already detected increase in trace-element concentrations indicates the sensitivity of the proposed approach and demonstrates its ability to detect early stages of accumulation before overt structural alterations become apparent.

Caution is needed when extending these observations beyond the specific model used here. Comparative data show that the relative contribution of the cornea, lens, retina and other ocular tissues to trace-element storage differs markedly among vertebrate groups, and that species display distinct ocular mineral profiles, as demonstrated in classical and more recent surveys [6,43]. Studies of bone, liver and other tissues in wild and domestic animals further indicate that growth rate, feeding strategy, housing conditions and lifespan substantially influence both the degree and the pattern of trace-element accumulation [57,58,59].

In order to account for these interspecies differences and assess the practical applicability of the proposed approach in intensive livestock production, it is important to consider the model species in the context of its production environment. Commercial rabbit and poultry production share several key structural features, including controlled indoor environments, high stocking densities and standardized compound feeds with premixed mineral supplements. These similarities make rabbits a suitable exploratory model for evaluating the general feasibility of corneal monitoring under intensive production. At the same time, it is likely that absolute corneal copper and iron ranges, as well as acceptable thresholds, differ between species and between extensive and intensive husbandry systems. This means that species-specific calibration will be required before broader application.

Several constraints of this study should be considered when interpreting the findings. The work was conducted within a single production setting with one genetic background and one type of compound feed, and the sample size was modest. Only copper and iron were quantified in corneal tissue, and no parallel measurements were obtained from blood, edible tissues or other ocular compartments, which means that direct cross-matrix comparisons are not yet available. The design was cross-sectional, so information on the temporal dynamics of corneal accumulation, including potential age-related or seasonal influences, could not be derived. Individual feed intake was not monitored, and contact with metallic surfaces was not quantified, which may have contributed to unexplained variability.

Even under standardized feed formulations, trace-metal status is determined not only by dietary concentration but also by actual intake and bioavailability, both of which are modulated by the animal’s homeostatic adaptation to trace-metal supply [45]. The chemical form of trace minerals and their relative bioavailability differ substantially between inorganic and organic sources used in production feeds and supplements, which can alter the efficiency of copper and iron absorption and their subsequent distribution among body compartments under otherwise similar rations [46]. For copper in particular, livestock studies emphasize that supplementation strategies must balance adequacy against the risk of excessive hepatic loading, demonstrating that tissue deposition can increase under sustained oversupply even in the absence of overt clinical toxicity [47]. In rabbits, dietary copper supplementation has been shown to markedly increase hepatic copper concentrations, indicating that sustained differences in net copper intake can translate into measurable tissue-level changes [48]. Similarly, controlled feeding studies in Rex rabbits report that graded dietary iron supplementation increases body iron stores and alters circulating iron-related indices, supporting the expectation that prolonged iron supply influences systemic status and tissue distribution [49]. Therefore, to improve the interpretability of corneal measurements as indicators of nutritional trace-element exposure, future validation studies should quantify copper and iron concentrations in specific feed batches and water sources, document the form and dose of mineral supplements, and, where feasible, record individual- or pen-level feed intake. This would allow corneal values to be linked to net mineral intake rather than to feed formulation alone [60,61].

It can be assumed that the elevated levels of iron and copper observed in some corneal specimens may be driven not only by internal metabolic factors but also by external influences. In particular, under cage-based housing conditions, rabbits may occasionally contact metallic components of the enclosure. Repeated microtrauma and material abrasion can result in the formation of iron-containing microparticles, which may adhere to the epithelial surface and penetrate into the superficial stromal layers. Experimental evidence supports the plausibility that iron-containing particulate material can traverse corneal barriers, as nano-sized iron particles have been shown to penetrate the cornea [62]. In addition, in vivo confocal microscopy studies of metallic corneal foreign body injuries report detectable metal deposits accompanied by tissue-level responses, consistent with the persistence of metallic particles within corneal layers [63]. Accordingly, in the absence of stringent contamination-control procedures, corneal measurements may partially reflect exogenous, surface-associated metal contributions in addition to endogenous accumulation. Future validation studies should therefore incorporate standardized surface decontamination and compartment-focused dissection, as well as established trace-element contamination control practices, including the use of pre-checked or acid-washed plasticware, protection from airborne dust, and routine analysis of procedural blanks processed alongside biological samples [64,65].

Earlier reports [66,67,68] describe similar cases of exogenous iron and copper accumulation in the cornea and other ocular structures, both in experimental models and in clinical settings, which may reflect mechanisms comparable to those observed in our study.

At present there is very limited information on how corneal trace-element concentrations relate quantitatively to levels in blood, internal organs and other matrices that are routinely used for monitoring in animal production and to our knowledge no systematic cross matrix calibrations have been reported in livestock. Studies that have profiled metals in the eye have typically focused on ocular tissues alone without concurrent sampling of systemic compartments [6], whereas investigations of dietary or environmental exposure in farm and wild animals have concentrated on bone, liver, kidney and muscle [30,31].

The present study should therefore be viewed as a proof of concept that demonstrates measurable variation in copper and iron in corneal tissue under commercial-type conditions rather than as a direct substitute for established matrices. Future work that combines corneal sampling with parallel measurements in blood and edible tissues, across different feeding regimens and species, will be required to determine whether robust quantitative relationships can be defined and whether corneal thresholds relevant to animal health and food-chain safety can be established.

## 5. Conclusions

The obtained data demonstrate the feasibility and proof-of-concept potential of the cornea as a biological material for assessing trace-element load in agricultural animals kept under commercial production conditions. Atomic absorption analysis revealed variability in stromal copper and iron content, including cases of elevated concentrations that were not accompanied by histologically detectable alterations. These findings indicate measurable inter-individual variation under standardized husbandry conditions rather than validation of a definitive biomarker.

The use of the eyeball as a research object offers several practical advantages. The organ is not included in the list of food products, can be easily removed during slaughter, and allows for standardized sampling for subsequent analysis. In this context, corneal analysis is not proposed as a replacement for established in vivo matrices, but may be considered as a complementary post-mortem matrix for monitoring chronic trace-element exposure, including applications in veterinary oversight and optimization of feeding strategies.

Further validation is required to support broader application of this approach. Future studies should combine corneal sampling with concurrent measurements in blood and edible tissues and incorporate defined feeding or exposure regimens, enabling the development of robust calibration strategies and meaningful decision thresholds for species- and production-specific interpretation.

## Figures and Tables

**Table 1:** Calibration solutions and detector signal levels. The KVANT.Z spectrometer applies inverse Zeeman background correction. The analytical absorbance signal A is calculated as the difference between total and background absorbance components measured under different magnetic field conditions. The values in this table report the peak amplitude of the corrected absorbance pulse A(t) used for calibration. a.u. indicates arbitrary units, i.e., instrument-reported signal intensity values used for calibration rather than concentration units.

Copper, µg/dm^3^	Analytical Absorbance Signal A, Peak Amplitude, a.u.	Iron, µg/dm^3^	Analytical Absorbance Signal A, Peak Amplitude, a.u.
0	0.01599	0	0.11405
1.0000	0.02312	20.000	0.34771
5.0000	0.05489	50.000	0.43915
10.000	0.09055	75.000	0.62895
20.000	0.16859	100.000	0.70571
40.000	0.36705	-	-

**Table 2:** Mean concentrations of copper and iron in the corneal stroma of laboratory rabbits. Values are presented in µg/g (native wet weight). For copper (copper), n = 22; for iron (iron), n = 21 after exclusion of the extreme value. Data are expressed as mean ± standard deviation. The full dataset, including the outlier, is presented in Supplementary Figure S1.

	Copper, μg/g (n = 22)	Iron (n = 21), μg/g
Mean ± SD	0.93 ± 0.46	0.78 ± 0.32
Range	0.19–2.1	0.21–1.44

## Data Availability

No new data were created or analyzed in this study. Data sharing is not applicable to this article.

## Supplementary Materials

The following supporting information can be downloaded at: https://www.mdpi.com/article/10.3390/metabo16030177/s1, Figure S1: Distribution plots of corneal iron concentrations in laboratory rabbits including the extreme outlier. Values are expressed in µg/g (native wet weight). (a)—histogram of distribution excluding the ex-treme value; (b)—boxplot excluding the extreme value. The distribution shows slight asymmetry and approximates normality.
