# Supplementary figures and images for: An Initial Assessment of Rabbit Cornea as a Biomarker of Trace-Element Load in Commercial Animal Production
Source: Metabolites. 2026 Mar 7;16(3):177. doi: 10.3390/metabo16030177 (PMC13028089; doi:10.3390/metabo16030177)

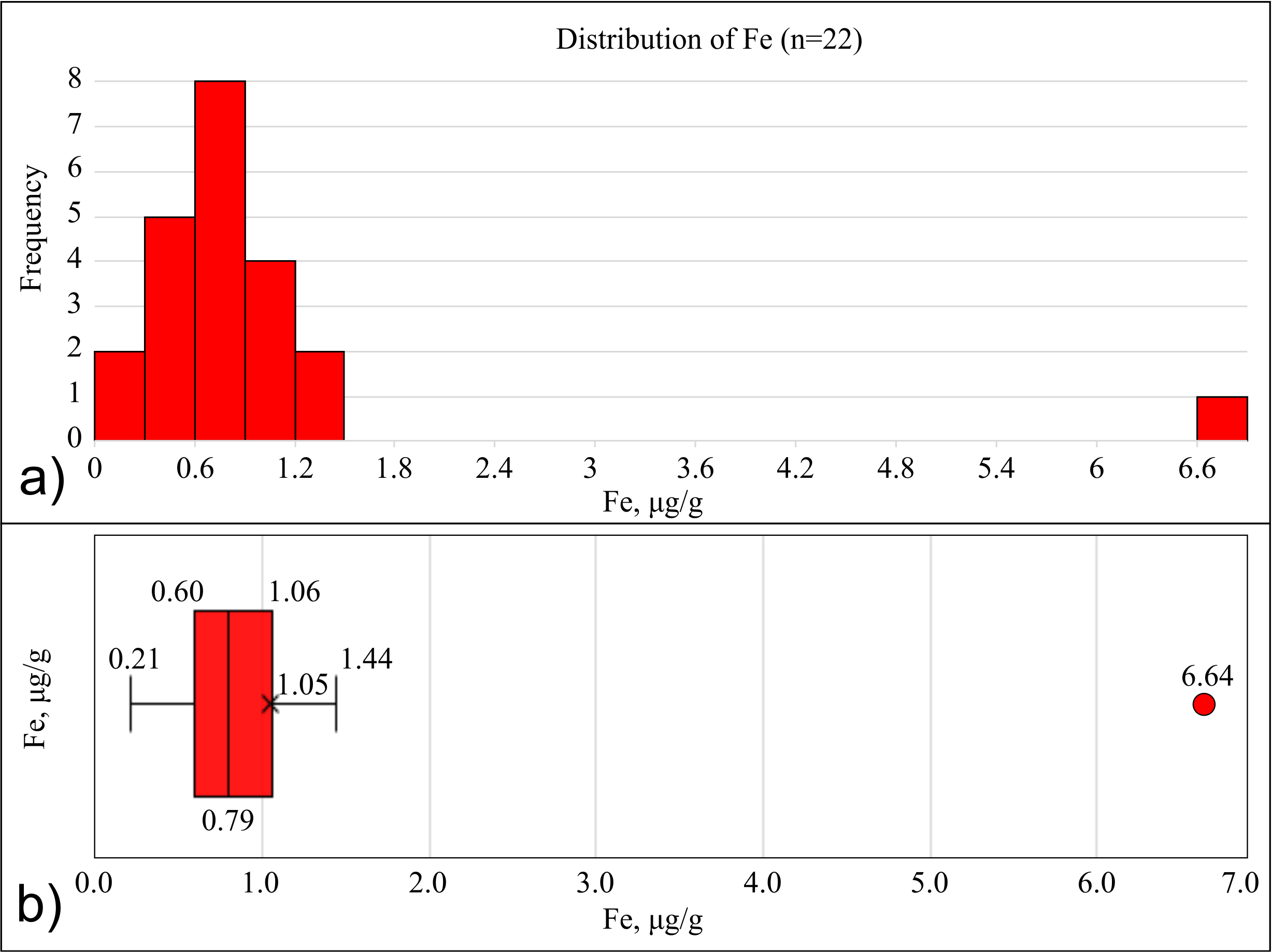

Supplement: Supplementary file 1 [file metabolites-16-00177-s001.zip › metabolites-4038294-supplementary.png]
